# Sports cardiology: A glorious past, a well‐defined present, a bright future

**DOI:** 10.1002/clc.24112

**Published:** 2023-09-12

**Authors:** Flavio D'Ascenzi, Gian L. Ragazzoni, Alex Boncompagni, Luna Cavigli

**Affiliations:** ^1^ Department of Medical Biotechnologies, Division of Cardiology University of Siena Siena Italy

**Keywords:** personalized exercise prescription, preparticipation screening, sports cardiology, sudden cardiac death

## Abstract

The attention towards sports cardiology has dramatically grown after the introduction of preparticipation screening and the need for specific education on electrocardiogram interpretation in athletes, given the differences between athletes and the general population. The present article stresses the need for specific skills, knowledge, and clinical expertise in sports cardiology, which are essential for appropriately screening competitive athletes to prevent sudden cardiac death and avoid overdiagnosis. However, disqualification from sports competitions may lead to sports inactivity, and athletes may enter a gray zone where little or no information is provided about what they can or cannot do to stay active. However, modern sports cardiology cannot neglect the patient's needs and the importance of the safe practice of regular exercise. In this context, the personalized exercise prescription plays a crucial role in the core curriculum and the clinical activity of professionals involved in sports cardiology programs. Given its specificities, sports cardiology requires a formal education plan for medical school students and all residents. Additional education and practice are required for young colleagues who want to focus their professional lives on sports cardiology. The future directions of emerging modern sports cardiology should not neglect the importance of a scientific community that works together, designing multicenter international outcomes‐based research to address the many remaining areas of uncertainty.

## Graphical abstract

The figure summarizes the dynamic process of sports eligibility and disqualification in competitive athletes, highlighting the role of a tailored personalized exercise prescription for athletes disqualified from sports competitions and sedentary individuals to counteract the negative effects of a sedentary lifestyle.

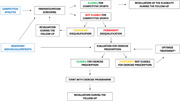

## 1 INTRODUCTION

Since the ancient Greek era, routine exercise has been considered a real medicine for body health. The Thracian physician Herodicus (5th century BC), one of Hippocrates' teachers, is widely considered the father of sports medicine. Herodicus and Hippocrates studies demonstrated the benefits of exercise for the whole body, but that century is also known for the first famous sudden cardiac death (SCD) in sport. Some research reported that Philippides died after a run to announce the victory of the Greek army against the Persians.^1^

The interest in sports‐related cardiovascular deaths started to grow when Jacob Mendez Da Costa, during the American Civil War, described a syndrome caused by the extreme physical efforts of long military campaigns. He called it “soldier's heart,”^2^ which was later modified to “athlete's heart,” a largely debated and studied subject still nowadays. Subsequently, sports cardiology was presented as a subspecialty of cardiology and sports medicine in April 1978, when the first Sports Cardiology International Congress was held in Rome. Italy was also the first country to have a sports cardiology society, founded in Rome in March 1981, aiming “to provide the guidance for participation in exercise and sport, including elite recreational and rehabilitation exercise.”^3^ In 1989, the Italian Society of Sports Cardiology and the Italian Federation of Sports Medicine published the first sports eligibility and disqualification recommendations. The European Association of Preventive Cardiology (EAPC) was officially created and registered as a branch of the European Society of Cardiology (ESC) on August 30, 2004, integrated with a Sports Cardiology and Exercise Section in 2005. Also, the American College of Cardiology (ACC), in 2011, developed the Exercise and Sports Cardiology Section. Several consensus documents have been published to support physicians in appropriately interpreting athlete's clinical data during the preparticipation screening (PPS) of professional or unprofessional individuals practising sports. This intense clinical activity culminated in the publication of international recommendations for electrocardiogram (ECG) interpretation in athletes,^4^ in the Italian Cardiological Guidelines (COCIS) for Competitive Sports Eligibility in athletes with heart disease,^5^ and in the more recent 2020 ESC guidelines on sports cardiology and exercise in patients with cardiovascular disease.^6^

## 2 PREPARTICIPATION SCREENING

The attention towards sports cardiology has dramatically grown after the introduction of the PPS and the need for specific education on ECG interpretation in athletes, given the differences between athletes and the general population. The PPS is a medical evaluation conducted on athletes before sports competitions aimed to detect a clinically silent cardiac condition that may increase the risk of SCD, or that may worsen with intensive exercise.^7^ It may be periodical (e.g., annually) or once in a lifetime. The rationale behind the PPS is that young adults who participate in competitive sports tend to exercise to their limits, thus increasing the risk of SCD, particularly when they are unaware of suffering from a cardiac disease predisposing to life‐threatening ventricular arrhythmias.^8^

The interest for a medical evaluation in competitive athletes before sports competitions started in Italy in the 50s. The PPS was established by the Italian government in 1982, with an annual screening that was mandatory by law in the entire nation for all the athletes who would like to compete, irrespective of their professional or nonprofessional status. The screening is conducted by sports medicine physicians for the program and includes history, physical examination, 12‐lead resting ECG, and exercise testing or an ECG‐monitored step test.^9^

Currently, a PPS is also enforced in the United States (history and physical examination only)^10^ and Israel and is advised by the European Society of Cardiology (ESC)^7^ and the Association of European Paediatric Cardiology (AEPC).^11^ Furthermore, although a PPS is not mandatory in most countries, several major sports organizations (e.g., FIFA, UEFA, and NBA) either recommend or enforce a cardiovascular evaluation that includes at least history, physical examination, and 12‐lead resting ECG.^7^, ^12^, ^13^

The evidence to support the enforcement of a PPS is based mainly on a study conducted by Corrado et al.^14^ in the Veneto region: the authors showed an 89% reduction in mortality rate after introducing the mandatory PPS in Italy. The arguments against the PPS are that, to date, other studies have not reproduced the results of the Italian experience. The Israeli experience, where a mandatory PPS with a 12‐lead resting ECG was introduced in 1997, did not reduce the mortality rate.^15^, ^16^ Furthermore, a study by Maron et al.^16^ did not show any difference in death rates of young athletes from the Veneto region (screened with the inclusion of the 12‐lead resting ECG) and Minnesota (screened with history and physical examination only), although the two populations have similar demographic characteristics. However, two major limitations should be considered when interpreting the data from these studies: the Israeli paper did not count the total number of participants in sports competitions; this data was an estimation. Moreover, in the American study, the information on the number of deaths during the competition was derived from insurance claims and media sources, likely underestimating the real number.

A further debated point is whether to include a 12‐lead resting ECG in the PPS: the ACC recommends history and physical examination only,^10^ and the ESC and AEPC recommend the inclusion of a 12‐lead resting ECG.^7^, ^11^ The rationale behind including the ECG is the significant increase in sensitivity for identifying cardiac conditions that may lead to SCD (from 20% to >90%).^17^, ^18^ Although the upside is a proven increase in sensitivity given by the ECG, on the other hand, it has imperfect specificity. In the last decades, to better account for the physiological training‐induced electrical adaptation in athletes, the proposals of new specific interpreting algorithms have reduced the presence of false positive results, and the current recommendations for ECG interpretation in athletes have demonstrated a good performance in this specific setting.^19^, ^20^, ^21^, ^22^, ^23^, ^24^

Therefore, according to the recommendations by several scientific societies and sports federations, the current approach is to screen competitive athletes with a comprehensive medical evaluation, including a 12‐lead resting ECG. Beyond what the current guidelines recommend, physicians involved in the PPS programs also include echocardiography to improve further the detection of cardiac diseases at risk of life‐threatening events.^25^

### 2.1 Exercise prescription in athletes disqualified from competitive sports

Given the importance of a comprehensive cardiovascular evaluation before participating in recreational and competitive sports, we must consider that the final diagnosis of a cardiac disorder usually leads to disqualification from sports practice.^6^, ^14^, ^26^ This may cause physical inactivity, a well‐recognized leading risk factor for overweight, obesity, diabetes, ischemic heart disease, cancer, chronic conditions, and ultimately all‐cause mortality, and major cardiovascular events.^27^ Conversely, physically active adults have a reduced risk of all‐cause and cardiovascular mortality, cancer, fractures, functional limitation, cognitive decline, and depression.^27^ To date, athletes who are disqualified from competitions often enter a gray zone where little or no information is provided about what they can or cannot do to stay active, and physicians are often reluctant to provide guidance due to the lack of data and limited confidence with exercise prescription in patients with cardiovascular disorders.^28^ Consequently, clinicians and patients feel the need to find the right balance in the dichotomy between competitive sports and a sedentary lifestyle.

Modern sports cardiology cannot neglect the patient's needs and the importance of the safe practice of regular exercise. In this context, the personalized exercise prescription plays a crucial role in the core curriculum and the clinical activity of professionals involved in sports cardiology programs. Indeed, a tailored exercise prescription requires specific clinical expertise and should consider individual characteristics, medical history and drugs, individual response to exercise, and an accurate evaluation of the clinical status and the phase of the disease.^28^

The exercise prescription is based on the so‐called “FITT‐VP” model (frequency, intensity, time, type, volume, and progression). Frequency is the number of sessions/week; Intensity is the rate of energy expenditure during exercise; Time represents the duration of a training program in weeks, training session times/day, and duration of training sessions in hours; Type of exercise includes endurance training (running, cycling, swimming, walking, etc.), strength or resistance training, respiratory, flexibility, and balance exercises^6^, ^29^; Volume indicates the total amount of exercise per week while Progression how is the program advanced.

While the prescription of frequency, time, and type of sport is more intuitive, the methodology to determine exercise intensity (EI) aimed at prescribing exercise is still debated.^30^ EI can be determined through subjective methods (i.e., Borg scale and the talk test) and objective methods (i.e., the percentage of maximal heart rate [HR] or HR reserve identified by exercise testing and the percentage of VO_2_max identified by cardiopulmonary exercise test [CPET]).^6^, ^30^, ^31^ The CPET gives the unique opportunity to identify the ventilatory thresholds (VTs), the first (VT_1_) and second (VT_2_) ventilatory thresholds, which represent the most reliable method to identify the correct intensity of aerobic exercise.^30^, ^32^ Indeed, the range‐based methods could misclassify EI, particularly in cardiac patients with a consequent over‐or under‐estimation of the intensity; therefore, the ventilatory threshold–based method is strongly recommended to prescribe an appropriate level of EI and to reduce the potential risk of a wrong intensity.^30^, ^31^, ^33^ Given the limited availability of CPET and its cost, lactate testing represents a valid method that may easily identify the first and second thresholds by determining lactate levels in the blood via withdrawal from the ear lobe and subsequent analysis of the lactate‐intensity curve. The first lactate or aerobic threshold corresponds to 2 mmol/L, and the second lactate or anaerobic threshold to 4 mmol/l.

Once an exercise program is prescribed, the patient must be followed over time and reevaluated.

Taking into account how important it is to manage the athletes in terms of sports practice after their disqualification, it is crystal clear that the evaluation of clinicians involved in the sports cardiology programs does not end with the disqualification of the athlete from sports competition but is completed by taking complete care of the newly diagnosed patient, giving him or her the unique opportunity of a new sports life through a personalized exercise prescription that aims to promote exercise therapy with a relative margin of safety. An informed decision shared with the patient is crucial, also taking into account that the exercise prescription programs, unfortunately, do not confer immunity from SCD to our patients, and an accurate self‐monitoring of symptoms during or after exercise sessions is essential.

The central illustration summarizes the dynamic process of sports eligibility and disqualification in competitive athletes, highlighting the role of a tailored personalized exercise prescription for athletes disqualified from sports competitions and sedentary individuals to counteract the negative effects of a sedentary lifestyle (see Central Illustration 1).

**Central Illustration 1:**
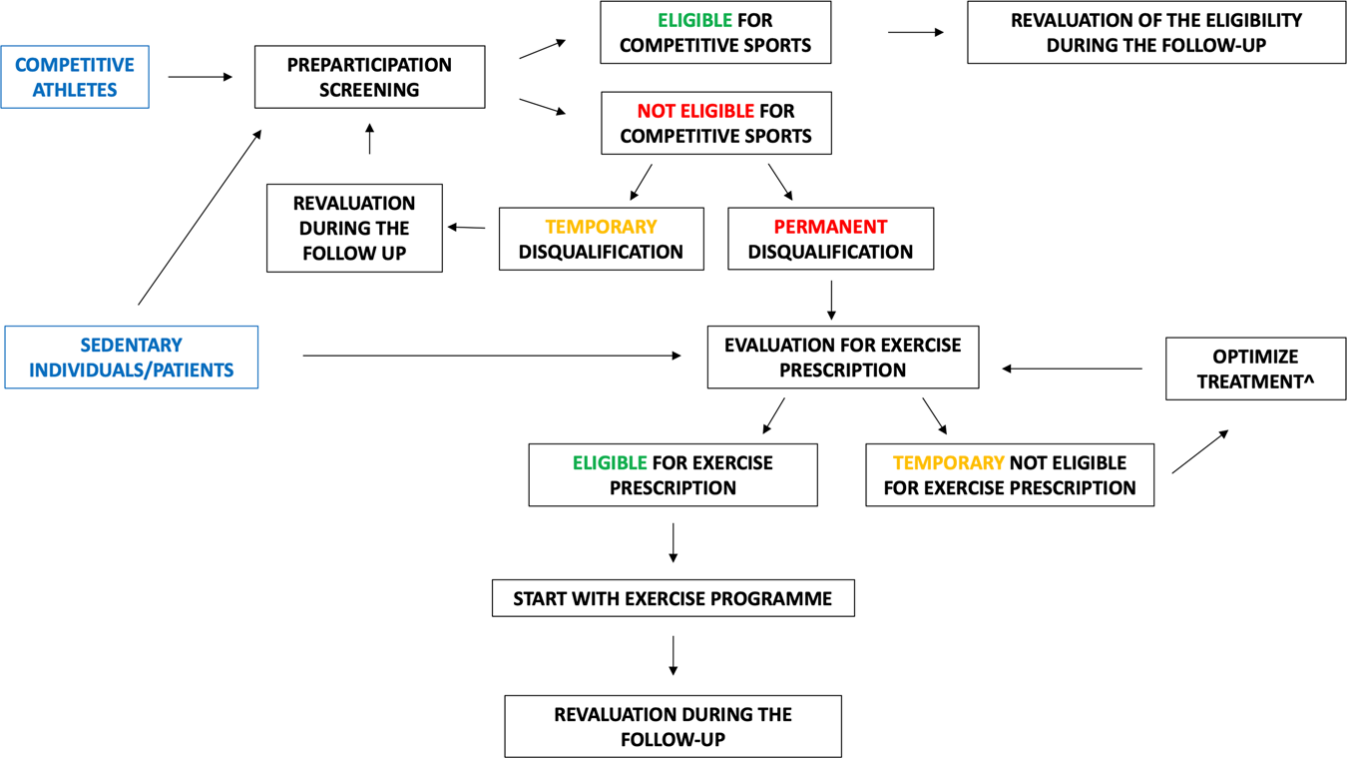
Competitive athletes should undergo comprehensive preparticipation screening, including a 12‐lead resting electrocardiogram. In case of a permanent disqualification, it is of paramount importance to evaluate the subject for a tailored exercise prescription. The prescription should include a comprehensive clinical evaluation, culminating in a personalized prescription with a specific target heart rate for aerobic exercise and detailed information about the intensity, type, volume, and frequency of exercise per week, including resistance conditioning (with a personalized intensity). In case of a clinical risk deemed to be excessively high and the impossibility to temporarily practice exercise, the patient should be reevaluated for an exercise program after the implementation of the medical therapy (e.g., after reducing a significant left ventricular outflow tract obstruction in patients with hypertrophic cardiomyopathy). The exercise program should start gradually, and a routine revaluation after the first months of exercise is essential to evaluate the adaptation to the exercise program, reevaluate the aerobic and anaerobic thresholds, and objectively and subjectively assess the program results. Exercise prescription should also be considered in sedentary individuals who do not want to practice competitive sports but are interested in a personalized training program for primary or secondary prevention. ^e.g., reduction of left ventricular outflow tract obstruction in patients with hypertrophic cardiomyopathy.

### 2.2 Sports cardiology: A specific core curriculum

The increased trend in the prevalence of obesity and diabetes mellitus that has tripled in Europe and the small changes in the prevalence of cardiovascular disease major components, ischemic heart disease, and stroke have changed the way of thinking of physicians.^34^ The scientific and clinical community has started a transition from predominantly treatment to prevention of cardiovascular disorders.^35^ Therefore, given the specific requirements in the field of cardiovascular prevention, the EAPC has proposed a specific core curriculum for preventive cardiologists in collaboration with the task force of the ESC Core Curriculum for Cardiologists.^36^ It recognized that preventive cardiology requires knowledge and skills that go beyond the basic requirements of general cardiology, and additional training is necessary. In the core curriculum published by the EAPC, sports cardiology has a relevant role and is considered among the domains and competencies required of physicians involved in primary and secondary prevention.

The exponential rise in knowledge and the growing demand for expertise in sports cardiology dictates the need to systematically structure the knowledge base of sports cardiology into a detailed curriculum. Accordingly, a specific core curriculum for a European Sports Cardiology qualification was proposed in 2013 by the EAPC.^37^ The sports cardiology syllabus proposed in this core curriculum includes principles of cardiac evaluation, prevention strategies of SCD, sports eligibility and disqualification for athletes with cardiac abnormalities, and cardiac rehabilitation in athletes with a cardiac diagnosis.

Subsequently, in 2017, the American College of Cardiology published a consensus document focused on a sports cardiology core curriculum for providing cardiovascular care to competitive athletes and highly active people, which defined the essential skills necessary to practice effective sports cardiology.^38^

All the teachers and residents in Cardiology, Sports Medicine, and Primary Care should read carefully and apply the general principles recommended by these papers, as Sports Cardiology is not a hobby but primarily represents a clinical need of our athletes and our patients.

## 3 CONCLUSIONS

Sports cardiology has evolved in the last few decades from the vision of a few enlightened pioneers to a clinical entity with specific requirements. As a subspeciality recognized worldwide, sports cardiology requires specific skills, knowledge, and clinical expertise, which are essential for appropriately screening competitive athletes to prevent SCD and avoid overdiagnosis. Furthermore, modern sports cardiology must also focus on the tailored prescription of exercise for athletes who are no longer eligible for sports competitions and for sedentary patients who need to become active. A formal education plan is required for medical school students and all residents. Additional education and practice are required for young colleagues who want to focus their professional lives on sports cardiology.

The future directions of the emerging modern sports cardiology should focus on some relevant and partially explored fields such as exercise prescription and validated ECG criteria for the population of children practising sport and should not neglect the importance of a scientific community that works together, designing multicenter international outcomes‐based research aimed to address the many remaining areas of uncertainty.

## AUTHOR CONTRIBUTIONS

All authors contributed to writing and reviewing the manuscript.

## CONFLICT OF INTEREST STATEMENT

The authors declare no conflict of interest.

## Data Availability

Data availability statements are not applied, given that original data were not presented in this manuscript.
